# Deep Eutectic Solvent-Based Extraction Optimization, Structural Characterization, and Alleviating Effects of *Tremella fuciformis* Polysaccharides on Ulcerative Colitis

**DOI:** 10.3390/foods15122207

**Published:** 2026-06-18

**Authors:** Zhenhua Fan, Qiuyun Li, Weiliang Wu

**Affiliations:** 1 Food Safety and Health Research Center, Guangdong Provincial Key Laboratory of Tropical Disease Research, School of Public Health, Southern Medical University, Guangzhou 510515, China; 15806021231@163.com; 2 Guangdong Medical Association, Guangzhou 510180, China; leeqy1218@163.com

**Keywords:** *Tremella fuciformis* polysaccharides, deep eutectic solvent, extraction optimization, structural characterization, ulcerative colitis, gut microbiota, intestinal barrier

## Abstract

*Tremella fuciformis* polysaccharides (TFPS) exhibit anti-inflammatory and gut-microbiota-modulating activities, but conventional extraction methods often show limited efficiency and may affect polysaccharide structural integrity. This study optimized a deep eutectic solvent (DES)-based extraction method with potential environmental advantages for TFPS, characterized the major purified fraction, and evaluated its effects in a dextran sulfate sodium (DSS)-induced experimental colitis model. Extraction parameters for the choline chloride–lactic acid DES system were refined through single-factor testing combined with response surface methodology. The purified fraction TFPS-1 was characterized by chromatographic, spectroscopic, methylation, and NMR analyses, and its biological effects were assessed in DSS-treated mice. Under the optimized conditions, the TFPS yield reached 33.09 ± 1.52%, representing a 77.6% increase compared with hot-water extraction. TFPS-1 was identified as a low-molecular-weight glucan mainly containing α-(1→4)- and β-(1→6)-linked glucose residues. In experimental colitis mice, TFPS-1 alleviated body weight loss, colon shortening, and histopathological injury; increased mucus secretion and barrier-related gene expression; reduced pro-inflammatory cytokines; increased IL-10; and partially adjusted gut microbiota composition. These results indicate that DES-based extraction is an efficient strategy for preparing TFPS and provide evidence that TFPS-1 may be further explored as a food-derived polysaccharide ingredient for intestinal protection in experimental colitis-related contexts.

## 1. Introduction

Ulcerative colitis (UC) is a recurrent inflammatory disorder affecting the colon, and its increasing prevalence has made long-term disease control a persistent clinical and public health concern [1,2]. UC pathogenesis involves not only mucosal inflammation but also epithelial barrier defects, immune dysregulation, luminal stimuli, and gut microbiota disturbance. Impaired barrier integrity increases mucosal exposure to microbial products and luminal antigens, thereby reinforcing inflammatory injury and disrupting intestinal homeostasis [3,4]. This barrier–inflammation–microbiota framework provides a rationale for evaluating food-derived polysaccharides as functional ingredients for intestinal protection in UC-related contexts [5,6,7,8]. However, such applications require more than general evidence of bioactivity; they require reproducible preparation, defined material properties, and validation using disease-relevant endpoints, as illustrated by recent polysaccharide studies in DSS-induced colitis models [9,10].

*Tremella fuciformis* is commonly used as a culinary fungus, and its polysaccharides (TFPS) are considered important bioactive constituents with potential value in functional food development [11,12]. Previous studies have reported diverse biological activities of TFPS, which are closely associated with molecular weight, monosaccharide composition, chain conformation, and fraction purity [13,14]. Some studies have suggested that TFPS may alleviate DSS-induced colonic injury through effects on inflammatory responses and epithelial barrier status, mucus production, gut microbiota, or microbial metabolites [15,16]. Nevertheless, TFPS is not a chemically uniform material, and its biological interpretation depends strongly on the extraction method, purification process, and degree of structural characterization [13,14,17,18]. Therefore, a preparation strategy that improves extraction efficiency while enabling structural clarification is necessary for evaluating TFPS as a functional food ingredient.

Deep eutectic solvents (DESs) have attracted attention as alternative media for polysaccharide extraction because their composition, polarity, viscosity, and water content can be adjusted for different biomass matrices [19]. Compared with conventional hot-water extraction, DES-based extraction may improve polysaccharide release and solubility while affecting molecular distribution and structural characteristics [20]. However, improved extraction yield alone is insufficient for functional food development unless the extracted polysaccharide is further purified, structurally characterized, and biologically validated [21]. Recent evidence also indicates that solvent selection and extraction parameters can substantially affect both extraction performance and material properties, supporting the need for systematic process optimization [22,23]. Thus, applying DES extraction to TFPS is meaningful when extraction optimization is linked with purified-fraction characterization and UC-related functional assessment.

Although previous studies have reported the intestinal protective effects of TFPS in DSS-induced colitis models [15,16], few studies have evaluated DES-extracted TFPS by combining extraction optimization, purification, structural characterization, and in vivo biological assessment of the same major polysaccharide fraction. Therefore, the main novelty of this work lies in its integrated framework, in which the major purified fraction TFPS-1 was obtained through optimized DES extraction, systematically characterized, and further evaluated in a DSS-induced experimental colitis model. The biological assessment included body weight, colon injury, histopathology, barrier-related gene expression, inflammatory cytokines, and gut microbiota. This study provides experimental evidence for the preparation, characterization, and intestinal protective evaluation of DES-extracted TFPS-1 in an experimental colitis context.

## 2. Materials and Methods

### 2.1. Materials and Chemicals

Fruiting bodies of *Tremella fuciformis* sourced from Gutian County, Fujian, China, were dried, milled into powder, and screened with a 60-mesh sieve before use. Dextran sulfate sodium (DSS) was supplied by Meilunbio Co., Ltd. (Dalian, China). Choline chloride (ChCl, ≥98%), DL-lactic acid (LA, ≥85%), phenol, concentrated sulfuric acid, anhydrous ethanol, chloroform, and n-butanol were purchased from Shanghai Macklin Biochemical Co., Ltd. (Shanghai, China). ELISA kits for TNF-α, IL-6, IL-1β, and IL-10 were obtained from Lianke Biotech Co., Ltd. (Hangzhou, China).

### 2.2. Preparation of Deep Eutectic Solvents

The ChCl-LA-based deep eutectic solvent (DES) was prepared by combining LA and ChCl at molar ratios ranging from 1:1 to 4.5:1. The water content was then adjusted to 30% (*w*/*w*) with deionized water. The mixture was stirred at 80 °C and 300 rpm for 2 h to form a clear homogeneous liquid. The prepared DES was cooled to room temperature before use.

### 2.3. Extraction of TFPS

Powdered *T. fuciformis* passing through a 60-mesh sieve was accurately weighed at 1.000 g and combined with DES in a 50 mL screw-cap glass tube according to the required liquid-to-solid ratio. The extraction process was performed in a thermostatic water bath under the preset experimental conditions. The extract was then centrifuged at 5000 rpm for 15 min, and the supernatant was separated. To precipitate polysaccharides, absolute ethanol was added until the ethanol concentration reached 75% (*v*/*v*), followed by incubation at 4 °C for 24 h [24]. After centrifugation, the precipitated material was dissolved again in deionized water and subjected to deproteinization by six rounds of Sevag treatment using chloroform/n-butanol (4:1, *v*/*v*) [25]. The treated solution was transferred into dialysis tubing with a 3.5-kDa molecular weight cutoff and dialyzed in deionized water for 72 h, during which the dialysis medium was refreshed periodically. Finally, the dialysate was freeze-dried to yield crude *T. fuciformis* polysaccharides. Total polysaccharide content was determined by the phenol–sulfuric acid method, using glucose for calibration [26].

TFPS yield was calculated as follows:

TFPS yield (%) = (C × V × D/m) × 100(1)

where C, V, D, and m represent the glucose-equivalent polysaccharide concentration (mg/mL), sample volume (mL), dilution factor, and dry weight of the initial *T. fuciformis* powder (mg), respectively.

### 2.4. Single-Factor Experiments

Single-factor experiments were conducted to evaluate the effects of extraction temperature (A), extraction time (B), liquid-to-solid ratio (C), and LA:ChCl molar ratio (D) on TFPS yield. During each experiment, only one extraction parameter was changed, whereas the remaining three parameters were maintained at fixed values.

To assess the effect of extraction temperature, A was set at 65, 70, 75, 80, 85, 90, 95, and 98 °C, while B, C, and D were fixed at 2 h, 30 g/g, and 3.5, respectively. For extraction time, B was set at 15, 30, 60, 90, 120, 150, 180, and 210 min, with A, C, and D maintained at 90 °C, 30 g/g, and 3.5, respectively. The liquid-to-solid ratio was evaluated by setting C at 15, 20, 25, 30, 35, 40, 45, and 50 g/g with A, B, and D maintained at 90 °C, 2 h, and 3.5. To investigate the influence of DES composition, D was adjusted to 1.0, 1.5, 2.0, 2.5, 3.0, 3.5, 4.0, and 4.5, while A, B, and C were kept at 90 °C, 2 h, and 30 g/g, respectively. Each experiment was performed in triplicate, and TFPS yield was used as the response.

### 2.5. Response Surface Methodology

The optimal extraction conditions were further determined by response surface methodology (RSM) based on the preliminary single-factor results. A Box–Behnken design with four variables and three levels was applied. TFPS yield (Y, %) was defined as the response value. The corresponding actual levels and coded values of each factor are shown in Table 1.

The experimental matrix was generated and analyzed using Design-Expert software, version 8.1.5 (Stat-Ease Inc., Minneapolis, MN, USA). The predicted optimal conditions were adjusted to practical operating values and validated by independent triplicate experiments.

### 2.6. Purification of TFPS

Crude TFPS was prepared as a 10 mg/mL aqueous solution and passed through a 0.45 μm membrane before chromatographic separation. The filtrate was loaded onto a DEAE-52 cellulose column (3.5 × 60 cm) pre-equilibrated with deionized water. Stepwise elution was performed with deionized water followed by NaCl solutions (0.1, 0.2, and 0.4 mol/L) at 1.0 mL/min. Fractions were collected at 10 mL per tube and monitored for carbohydrate content by the phenol–sulfuric acid method at 490 nm. The predominant fraction eluted with water was combined, dialyzed, freeze-dried, and designated TFPS-1. Further purification of TFPS-1 was performed on a Sephadex G-100 column (2.5 × 60 cm) using deionized water as the mobile phase at 0.8 mL/min. The main peak fraction was collected, dialyzed in deionized water for 72 h with periodic replacement of the dialysis medium, and finally lyophilized to obtain purified TFPS-1 for subsequent characterization and bioactivity assays [27].

### 2.7. Structural Characterization of TFPS-1

#### 2.7.1. Molecular Weight Determination

The molecular weight of TFPS-1 was determined by HPGPC using a Waters 1515 HPLC system equipped with a Waters 2414 refractive index detector, both from Waters Corporation (Milford, MA, USA). Separation was performed on three tandem polymer-based aqueous gel columns (8 mm × 300 mm) at 40 °C, with 0.05 mol/L NaCl as the mobile phase at 0.65 mL/min. A calibration curve was constructed using dextran standards (T-10, T-40, T-70, T-110, and T-200). TFPS-1 was dissolved in 0.05 mol/L NaCl at 5 mg/mL, centrifuged at 8000 rpm for 10 min, filtered through a 0.22 μm membrane, and injected at 30 μL.

#### 2.7.2. Monosaccharide Composition Analysis

The monosaccharide composition of TFPS-1 was measured by HPLC after TFA hydrolysis and PMP derivatization. Briefly, TFPS-1 was hydrolyzed with 2 mol/L TFA at 110 °C for 4 h, dried under nitrogen, and derivatized with PMP at 70 °C for 2 h. The derivatives were separated using a Thermo Dionex U3000 HPLC system (Thermo Fisher Scientific, Waltham, MA, USA) fitted with an Agilent ZORBAX Eclipse XDB-C18 column (4.6 × 250 mm, 5 μm; Agilent Technologies, Santa Clara, CA, USA). The mobile phase was acetonitrile/phosphate buffer (17:83, *v*/*v*), delivered at 0.8 mL/min, with the column temperature set at 30 °C, detection at 250 nm, and an injection volume of 10 μL. Monosaccharides were identified and quantified by comparison with authentic standards using the external standard method.

#### 2.7.3. Fourier Transform Infrared (FTIR) Spectroscopy

Fourier transform infrared spectroscopy was performed to characterize the functional groups of TFPS-1. The sample was prepared by mixing TFPS-1 powder with dry KBr at a ratio of 1:300 and pressing the mixture into a transparent pellet. Spectra were recorded on a Nicolet iS50 FTIR spectrometer (Thermo Fisher Scientific, Waltham, MA, USA) from 4000 to 400 cm^−1^ at 4 cm^−1^ resolution over 32 scans.

#### 2.7.4. Scanning Electron Microscopy (SEM)

The microstructure of TFPS-1 was analyzed by SEM. Lyophilized TFPS-1 powder was mounted on a copper stub with conductive adhesive and coated with a thin layer of gold. Images were obtained using a Sigma 360 SEM (Zeiss, Jena, Germany).

#### 2.7.5. Methylation Analysis

Glycosidic linkage patterns of TFPS-1 were elucidated by methylation analysis combined with GC-MS. Permethylation was performed by treating the sample with methyl iodide in a dimethyl sulfoxide/NaOH suspension. The permethylated product was recovered by extraction, hydrolyzed with 2 mol/L TFA at 121 °C for 90 min, reduced with NaBD_4_, and acetylated with acetic anhydride. The resulting partially methylated alditol acetates were passed through a 0.22 μm filter before GC–MS analysis. Separation was carried out on an Agilent 7890A-5977B system equipped with an HP-5MS capillary column, using high-purity helium as the carrier gas at 1.0 mL/min. A 1 μL aliquot was injected at a split ratio of 10:1, with the injector temperature maintained at 260 °C. The oven program started at 50 °C for 1 min, increased to 130 °C at 50 °C/min, then rose to 230 °C at 3 °C/min and was held for 2 min. Mass spectra were acquired under electron-impact ionization at 70 eV over an *m*/*z* range of 30–600.

#### 2.7.6. NMR Spectroscopic Analysis

For NMR analysis, 20 mg of TFPS-1 powder was dissolved in D_2_O and lyophilized three times to exchange labile protons. After drying, the sample was reconstituted in 0.5 mL of D_2_O supplemented with 0.05% TMSP as the internal reference. ^1^H, ^13^C, COSY, NOESY, HSQC, and HMBC NMR spectra were acquired on a Bruker AVANCE III 600 MHz spectrometer Bruker BioSpin GmbH, Rheinstetten, Germany at 298 K. Spectral processing and peak assignment were performed using MestReNova 14.0.

#### 2.7.7. Congo Red Assay

The possible triple-helix conformation of TFPS-1 was preliminarily evaluated using the Congo red assay. TFPS-1 solution (2 mL, 2.5 mg/mL) was mixed with an equal volume of Congo red solution (80 μmol/L). NaOH solution (4 mol/L) was then added stepwise to obtain final NaOH concentrations of 0, 0.1, 0.2, 0.3, 0.4, and 0.5 mol/L. After equilibration at room temperature for 10 min, each mixture was scanned from 400 to 600 nm using a UV-visible spectrophotometer (Thermo Fisher Scientific, Waltham, MA, USA). The maximum absorption wavelength was recorded at each NaOH concentration. Congo red solution treated under the same alkaline conditions without TFPS-1 was used as the control.

### 2.8. Animal Experiment

#### 2.8.1. Animals, Housing, and Experimental Treatment

Six-week-old male BALB/c mice weighing 18–22 g were purchased from Guangdong Yaokang Biotech Co., Ltd. (Guangzhou, China; animal production license No. SCXK (Yue) 2020-0054). The animal protocol was reviewed and approved by the Animal Ethics Committee of Southern Medical University (approval No. SMUL202408045), and all animal procedures followed the National Institutes of Health Guide for the Care and Use of Laboratory Animals.

Animals were maintained in a specific-pathogen-free (SPF) environment with regulated parameters: temperature 20–23 °C, relative humidity 40–70%, and a 12 h light/dark photoperiod. Animals were housed four per cage, and given ad libitum access to sterile water and standard rodent chow. Using a computerized randomization schedule, 60 animals were allocated to five groups after 7 days of acclimatization (*n* = 12 per group): CON, MOD, TFPS-L, TFPS-M, and TFPS-H.

**Figure 1:**
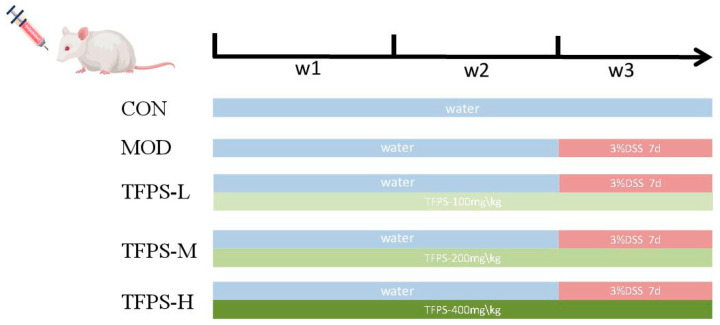
Experimental design of DSS-induced colitis and TFPS-1 intervention in mice.

As shown in Figure 1, the CON group received normal drinking water and daily gavage with 0.2 mL normal saline for 21 days. The MOD group received normal drinking water from days 1–14, followed by 3% DSS from days 15–21, with daily saline gavage. The TFPS-L, TFPS-M, and TFPS-H groups received TFPS-1 by daily gavage at 100, 200, and 400 mg/kg, respectively, for 21 days and were exposed to 3% DSS from days 15–21.

#### 2.8.2. Health Status Surveillance and Disease Activity Index Assessment

General health status, activity, food intake, stool consistency, and fecal bleeding were monitored throughout the experiment. Body weight and food intake were measured every 2–3 days. DAI was scored according to Table 2 by evaluating weight change, stool consistency, and fecal bleeding.

#### 2.8.3. Histopathological and AB-PAS Staining Analysis

Colon tissues were immediately immersed in 4% paraformaldehyde after collection, fixed overnight, dehydrated, embedded in paraffin, and sectioned at 5 μm. For H&E staining, sections were dewaxed, rehydrated, stained with hematoxylin and eosin, dehydrated, cleared, and mounted. Adjacent sections were subjected to AB-PAS staining to assess goblet cells and mucus secretion. Briefly, sections were dewaxed, rehydrated, stained with Alcian blue, oxidized with periodic acid, treated with Schiff reagent, counterstained with hematoxylin, dehydrated, cleared, and mounted. Images were acquired at identical magnification using a Nikon Eclipse Ci microscope coupled with a Nikon DS-U3 imaging system. Histological changes in H&E- and AB-PAS-stained colon sections were assessed descriptively by investigators blinded to the group allocation. The assessment included mucosal epithelial integrity, crypt architecture, inflammatory cell infiltration, goblet cell abundance, and mucus secretion. No quantitative histopathological scoring system was applied in this study.

#### 2.8.4. Measurement of Inflammatory Cytokines

Colon tissue samples were homogenized in RIPA lysis buffer supplemented with protease inhibitor, phosphatase inhibitor, and PMSF. Following ultrasonic disruption, the homogenates were centrifuged at 14,000 rpm for 15 min at 4 °C, and the supernatants were retained. Total protein levels were measured using the BCA assay. The protein levels of TNF-α, IL-6, IL-1β, and IL-10 were measured using commercial ELISA kits according to the manufacturer’s instructions. Optical density was measured using a microplate reader, and cytokine levels were normalized to total protein concentration.

#### 2.8.5. qRT-PCR Analysis

Total RNA was extracted from colonic samples with TRIzol isolation reagent, and the resulting concentration and purity were evaluated using a NanoDrop spectrophotometer (Thermo Fisher Scientific, Waltham, MA, USA). A commercial cDNA synthesis kit (Lianke Biotech Co., Ltd., Hangzhou, China) was employed to reverse transcribe 500 ng of RNA into complementary DNA. The synthesized cDNA was subsequently diluted and served as the template for quantitative real-time PCR amplification with SYBR Green I detection chemistry. Specific primer details are listed in Table S3. The thermocycling conditions comprised a 95 °C denaturation step for 30 s, then 40 cycles of denaturation at 95 °C for 5 s and annealing/extension at 60 °C for 30 s, concluding with a melting curve analysis. The reference gene β-actin was utilized for normalization, and relative transcript abundance was calculated according to the 2^−ΔΔCt^ method.

#### 2.8.6. Gut Microbiota Analysis

Colon contents were collected under sterile conditions and stored at −80 °C until analysis. For gut microbiota profiling, five samples per group were used. Microbial genomic DNA was obtained using a CTAB/SDS-based extraction procedure, and its integrity was checked by agarose gel electrophoresis. DNA that met the quality requirements was adjusted to 1 ng/μL and used for amplification of the bacterial 16S rRNA V4 region. The amplified products were subsequently purified, quantified, and combined at equal molar concentrations.

Sequencing libraries were constructed using Kinnex long-read sequencing technology and sequenced on the PacBio Revio platform using single-molecule real-time (SMRT) sequencing. Raw reads were demultiplexed according to barcode and primer sequences, and HiFi reads were quality-filtered using SeqKit v2.9.0. Taxonomic annotation was performed against the SILVA 138.1 database using the Mothur classifier. Representative sequences were aligned using MUSCLE v3.8.31, and all samples were rarefied to the minimum sequencing depth before downstream analyses.

Alpha diversity was evaluated using Simpson, Shannon, ACE, and Chao1 indices. Beta diversity was assessed based on weighted and unweighted UniFrac distance matrices, and non-metric multidimensional scaling (NMDS) was used to visualize differences in microbial community structure among groups. Group differences in beta diversity were assessed using PERMANOVA implemented with the Adonis function in R. Venn diagrams were used to display shared and group-specific ASVs. Microbial taxonomic composition was profiled at the phylum and genus levels. Differentially abundant taxa among groups were identified using linear discriminant analysis effect size (LEfSe), with a Kruskal–Wallis test followed by pairwise Wilcoxon tests and an LDA score threshold of 3.0. Where multiple pairwise comparisons were performed, *p*-value correction was applied when applicable.

### 2.9. Statistical Analysis

Data are presented as mean ± SD and were analyzed using GraphPad Prism 9.5 unless otherwise stated. For repeated-measurement outcomes, including body weight, food intake, and disease activity index (DAI), differences among groups over time were analyzed using two-way repeated-measures ANOVA, with treatment group and time as the main factors, followed by Tukey’s multiple-comparison test. For endpoint measurements, including colon length, organ indices, cytokine levels, and gene expression, normality and homogeneity of variance were assessed before statistical comparison. When these assumptions were met, differences among groups were analyzed using one-way ANOVA followed by Tukey’s post hoc test. For data that did not meet these assumptions, non-parametric tests were used. RSM analysis and gut microbiota analyses were performed as described in the corresponding sections.

## 3. Results

### 3.1. Optimization of DES-Based TFPS Extraction

#### 3.1.1. Single-Factor Experiments

**Figure 2:**
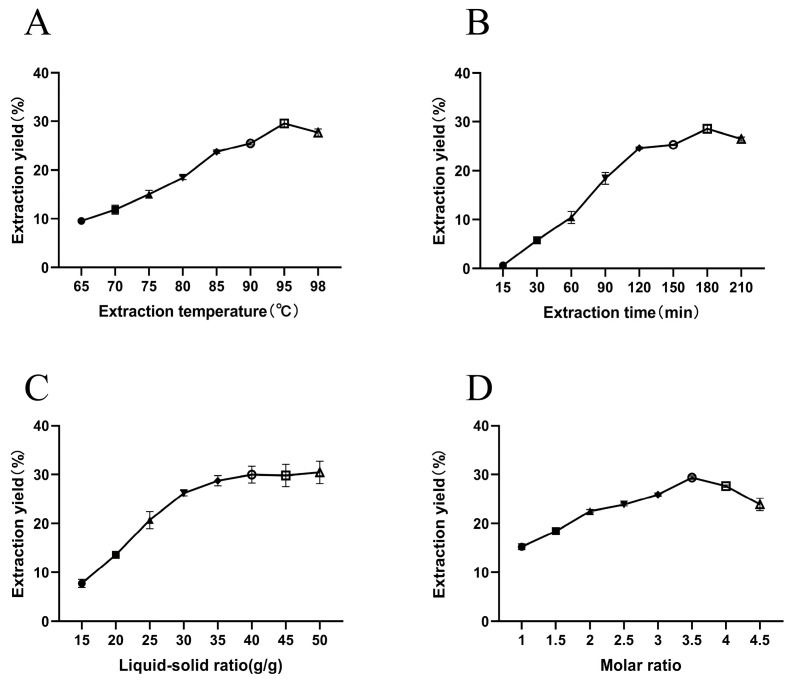
Effects of extraction parameters on TFPS yield. (**A**) Extraction temperature; (**B**) Extraction time; (**C**) Liquid-to-solid ratio; (**D**) LA:ChCl molar ratio.

Preliminary single-factor screening indicated that extraction conditions affected TFPS yield (Figure 2). TFPS yield increased with the adjustment of key extraction parameters within suitable ranges, but excessive levels led to either a plateau or a decrease in yield.

#### 3.1.2. Response Surface Methodology Optimization

Table S1 presents the Box–Behnken design matrix and corresponding TFPS yields. The data were fitted to a second-order polynomial model, yielding the following regression equation:

(2) $$\begin{array}{ll} Y= & 32.518+1.942A+1.037B-0.651C-0.546D+1.018\mathrm{AB}+0.038\mathrm{AC}-0.475\mathrm{AD}\\ & -0.365\mathrm{BC}+0.608\mathrm{BD}-0.420\mathrm{CD}-3.164A^{2}-0.994B^{2}-1.728C^{2}-0.968D^{2} \end{array}$$

where Y represents TFPS yield and A, B, C, and D coded values of extraction temperature, extraction time, liquid-to-solid ratio, and LA:ChCl molar ratio.

**Figure 3:**
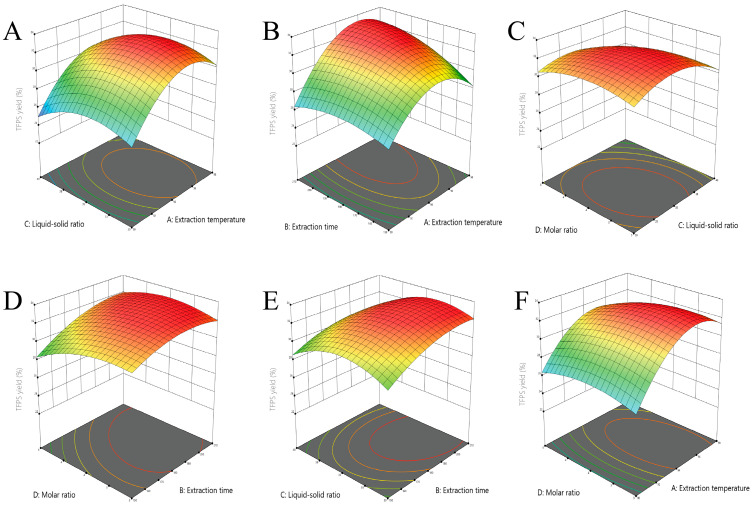
Response surface plots showing the interaction effects of extraction variables on TFPS yield: (**A**) Liquid-to-solid ratio and extraction temperature; (**B**) extraction time and extraction temperature; (**C**) LA:ChCl molar ratio and liquid-to-solid ratio; (**D**) LA:ChCl molar ratio and extraction time; (**E**) Liquid-to-solid ratio and extraction time; and (**F**) LA:ChCl molar ratio and extraction temperature.

As shown in Table S2, ANOVA confirmed the significance of the regression model, and the non-significant lack-of-fit result (*p* = 0.0938) indicated adequate model fitness for TFPS yield prediction. The R^2^ and adjusted R^2^ values were 0.9826 and 0.9625, indicating that the model accounted for a large proportion of the variability in TFPS yield. Among the tested factors, their relative effects followed the order: A > B > C > D. The response surface plot in Figure 3 illustrates the interaction between the conditions.

#### 3.1.3. Validation of Optimal Conditions

The model predicted the maximum TFPS yield of 33.13% under the following optimal conditions: 96.87 °C, 205.53 min, 34.67 g/g, and LA:ChCl molar ratio of 3.49. For practical operation, the conditions were adjusted to 95 °C, 210 min, 35 g/g, and LA:ChCl molar ratio of 3.5. The adjusted extraction conditions were validated in three independent replicate experiments, and the average TFPS yield was 33.09 ± 1.52%, which was consistent with the predicted value. Under identical conditions, hot water extraction yielded only 18.63 ± 2.15% by comparison. Thus, DES-based extraction increased TFPS yield by 77.6% compared with hot-water extraction.

### 3.2. Purification and Structural Characterization of TFPS-1

#### 3.2.1. Purification of TFPS-1

**Figure 4:**
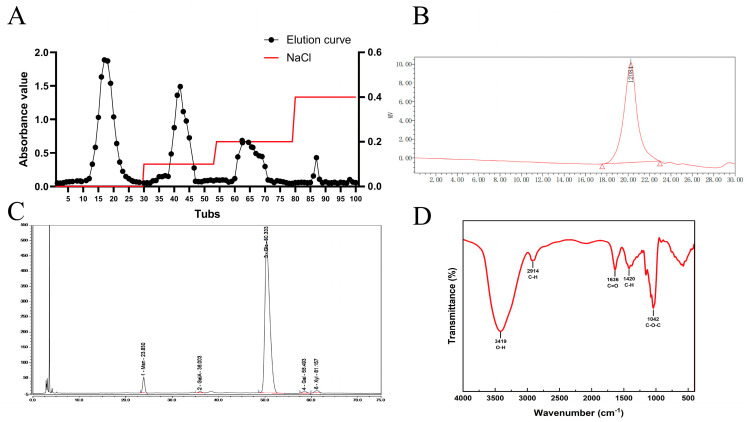
Purification and structural characterization of TFPS-1. (**A**) Elution profile of crude TFPS on a DEAE-52 cellulose column. (**B**) HPGPC chromatogram of purified TFPS-1. (**C**) HPLC chromatogram of TFPS-1 monosaccharide composition. (**D**) FTIR spectrum of TFPS-1.

Crude TFPS was separated by DEAE-52 cellulose column chromatography into four fractions (Figure 4A): TFPS-1, TFPS-2, TFPS-3, and TFPS-4 were obtained by elution with deionized water, 0.1, 0.2, and 0.4 mol/L NaCl solutions, respectively. TFPS-1 was the predominant carbohydrate-containing fraction and was further purified by Sephadex G-100 column chromatography, yielding a single symmetric peak, suggesting relative chromatographic homogeneity. The purity of TFPS-1 was expressed as the total carbohydrate content, which was determined to be 92.16 ± 0.59% using the phenol–sulfuric acid method.

#### 3.2.2. Molecular Weight and Monosaccharide Composition

HPGPC analysis showed that TFPS-1 had a weight-average molecular weight (Mw) of 16,999 Da, a number-average molecular weight (Mn) of 4918 Da, and a polydispersity index (Mw/Mn) of 3.46, indicating a relatively broad molecular weight distribution (Figure 4B).

Monosaccharide profiling showed that TFPS-1 consisted predominantly of glucose (93.34%), along with minor proportions of mannose (4.09%), xylose (1.15%), galactose (0.89%), and galacturonic acid (0.53%) (Figure 4C). The molar ratio of these monosaccharides was 100:4.38:1.23:0.95:0.57.

#### 3.2.3. FTIR Analysis

As shown in Figure 4D, the FTIR spectrum of TFPS-1 exhibited absorption bands typical of polysaccharides. A broad peak centered near 3419 cm^−1^ was ascribed to O–H stretching vibrations, and the signal at 2914 cm^−1^ reflected C–H stretching arising from methyl and methylene moieties. The band near 1636 cm^−1^ may be associated with bound water and/or C=O stretching vibrations. The signal at 1042 cm^−1^ was assigned to C–O–C stretching of glycosidic bonds. Moreover, the absorption near 890 cm^−1^ suggested the presence of β-glycosidic configurations.

#### 3.2.4. SEM Morphological Analysis

**Figure 5:**
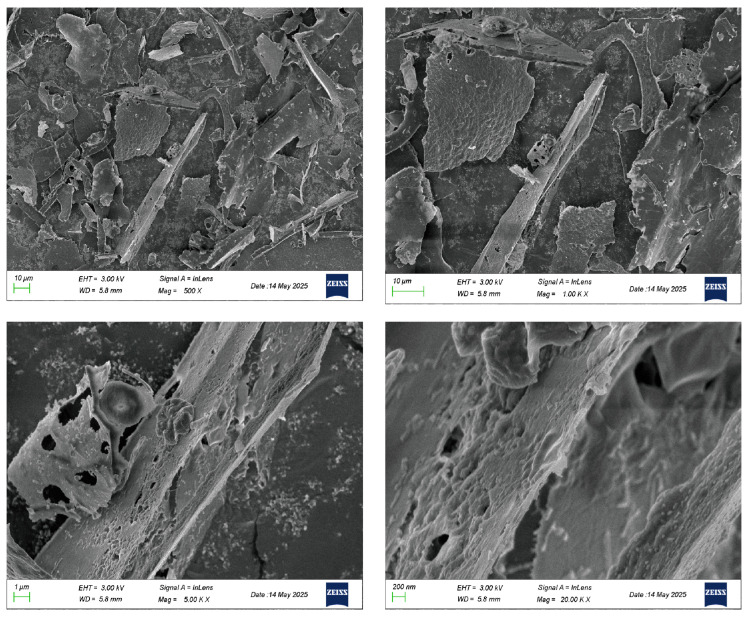
Scanning electron microscopy images of TFPS-1.

SEM images of TFPS-1 at different magnifications are shown in Figure 5. At low magnification (500× and 1000×), TFPS-1 exhibited an irregular sheet-like structure with uneven edges and a loose network formed by overlapping sheets. At medium magnification (5000×), the sheets showed a porous, sponge-like structure with irregularly sized pores. At high magnification (20,000×), the surface of the sheets had a fine fibrous or membranous texture with nanoscale granular protrusions.

#### 3.2.5. Methylation Analysis

Methylation analysis identified five major types of glycosidic linkages in TFPS-1 based on the characteristic PMAA derivatives and GC–MS fragmentation patterns (Table 3) [28]. The predominant linkages were →4)-Glcp-(1→ and →6)-Glcp-(1→, accounting for 45.12% and 39.18% of the total linkages, respectively. Terminal Glcp linkages represented 9.26%, while the branched residues →4,6)-Glcp-(1→ and →3,6)-Glcp-(1→ accounted for 2.25% and 4.19%, respectively. These findings indicate that TFPS-1 is a branched glucan mainly composed of 1→4 and 1→6 glycosidic linkages.

#### 3.2.6. NMR Spectroscopy

**Figure 6:**
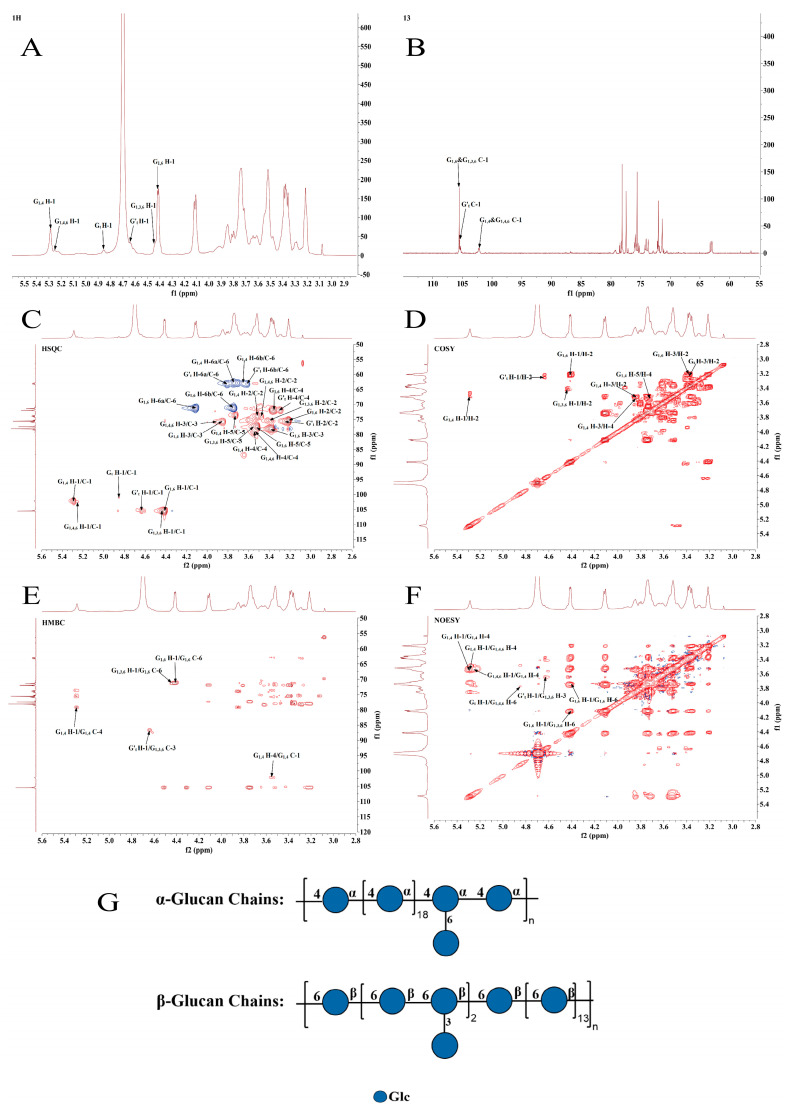
NMR characterization and proposed structural features of TFPS-1. (**A**) ^1^H NMR spectrum, (**B**) ^13^C NMR spectrum, (**C**) HSQC spectrum, (**D**) COSY spectrum, (**E**) HMBC spectrum, and (**F**) NOESY spectrum. (**G**) Proposed structural model of TFPS-1, showing an α-glucan chain mainly composed of α-(1→4)-linked Glcp residues with branches at O-6, together with a β-glucan chain mainly composed of β-(1→6)-linked Glcp residues with branches at O-3. Glc, glucose residue.

NMR spectroscopy was used to further elucidate the structural features of TFPS-1 (Figure 6) [29]. The ^1^H NMR spectrum showed overlapping signals in the carbohydrate region, while the ^13^C NMR spectrum displayed clearer anomeric carbon signals between δ 90 and 110 ppm. Based on the combined results of monosaccharide composition, methylation analysis, HSQC, and COSY spectra, six major glucosyl residues were identified: →4)-α-D-Glcp-(1→, →4,6)-α-D-Glcp-(1→, terminal α-D-Glcp, →6)-β-D-Glcp-(1→, →3,6)-β-D-Glcp-(1→, and terminal β-D-Glcp. Their chemical shift assignments are summarized in Table 4.

Downfield shifts in the NMR spectra confirmed the substitution positions. The C-4 signal moved downfield, consistent with O-4 substitution in the α-glucan units, and a pronounced C-3 downfield shift for →3,6)-β-D-Glcp-(1→ verified substitution at O-3. HMBC and NOESY correlations supported these linkages. Specifically, the α-glucan portion comprised an α-(1→4)-linked backbone featuring →4,6)-α-D-Glcp-(1→ branch points, where terminal α-D-Glcp residues were attached at O-6. The β-glucan portion consisted of a β-(1→6)-linked main chain carrying terminal β-D-Glcp branches at O-3 of →3,6)-β-D-Glcp-(1→ residues.

Taken together, TFPS-1 was proposed to be a branched glucan composed mainly of α-(1→4)- and β-(1→6)-linked glucose residues, with branches primarily connected through O-6 and O-3 positions. The proposed structural model is shown in Figure 6G.

#### 3.2.7. Congo Red Assay

**Figure 7:**
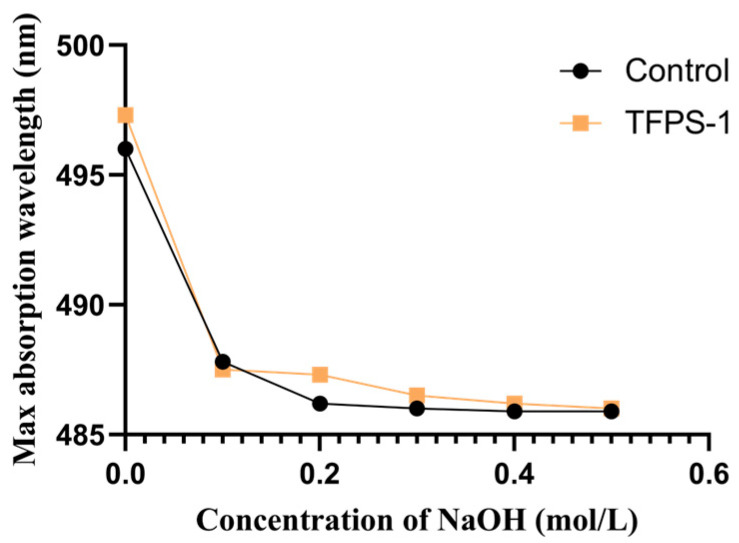
Congo red assay of TFPS-1 under different NaOH concentrations.

The possible ordered chain conformation of TFPS-1 was further examined using the Congo red assay. The maximum absorption wavelength of the TFPS-1–Congo red complex decreased as the NaOH concentration increased, following a pattern similar to that of the Congo red control (Figure 7). No distinct red shift was detected for the TFPS-1–Congo red complex within the tested NaOH concentration range. This result suggests that TFPS-1 may not possess a typical triple-helix conformation in solution.

### 3.3. Alleviating Effects of TFPS-1 on DSS-Induced Experimental Colitis in Mice

#### 3.3.1. Effects on General Health and Disease Activity

**Figure 8:**
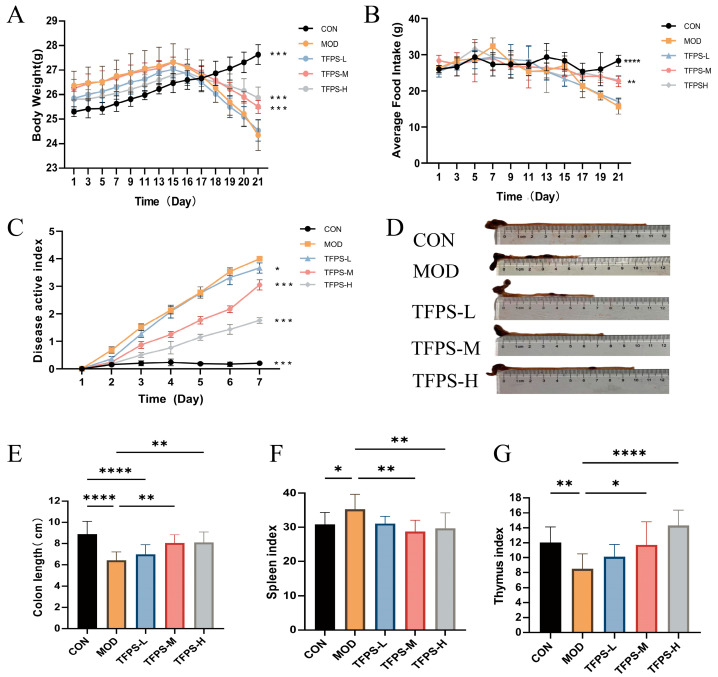
TFPS-1 attenuated DSS-induced disease manifestations and systemic changes in mice. (**A**) Changes in body weight. (**B**) Average food intake. (**C**) DAI. (**D**) Images of colon length. (**E**) Colon length measurement. (**F**) Spleen index. (**G**) Thymus index. Data are presented as mean ± SD (*n* = 12). * *p* < 0.05, ** *p* < 0.01, *** *p* < 0.001, **** *p* < 0.0001.

As shown in Figure 8A,B, DSS-exposed mice exhibited progressive body weight loss and reduced food intake during the later stage. TFPS-1 administration partially attenuated body weight loss and improved food intake, with stronger effects observed in the TFPS-M and TFPS-H groups. Consistently, DAI scores increased rapidly in the MOD group during DSS exposure, whereas TFPS-1 treatment reduced DAI scores, with more evident effects in the TFPS-M and TFPS-H groups (Figure 8C).

DSS exposure also resulted in marked colon shortening, as shown by representative colon images and quantitative measurements (Figure 8D,E). Compared with the CON group, the MOD group showed a marked reduction in colon length, while TFPS-1 treatment partially restored colon length, particularly in the TFPS-M and TFPS-H groups. These findings indicate that TFPS-1 mitigated the main manifestations of DSS-induced experimental colitis.

In addition, DSS treatment increased the spleen index and decreased the thymus index compared with the CON group (Figure 8F,G). TFPS-1 administration attenuated these changes, as evidenced by a lower spleen index and a higher thymus index compared with the MOD group. Collectively, these findings indicate that TFPS-1 partially attenuated the main manifestations of DSS-induced experimental colitis under the present experimental conditions.

#### 3.3.2. Effects on Colon Histopathology

**Figure 9:**
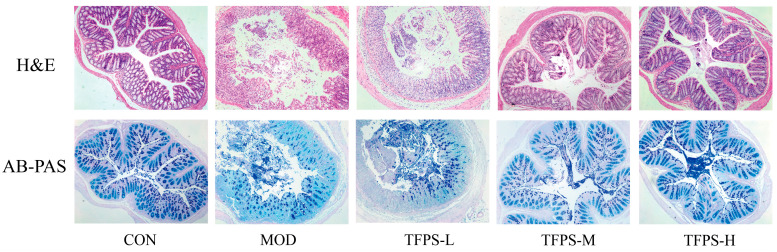
TFPS-1 improved colonic histopathology and restored mucus secretion in DSS-induced colitis mice. Images were captured at the same magnification.

H&E staining showed intact colonic mucosa, well-organized crypts, and no obvious inflammatory infiltration in the CON group (Figure 9). In contrast, DSS exposure caused severe colonic injury in the MOD group, characterized by epithelial disruption, crypt loss, mucosal erosion, and extensive inflammatory cell infiltration. These pathological alterations were mitigated by TFPS-1, especially in the TFPS-M and TFPS-H groups. The TFPS-M and TFPS-H groups showed improved epithelial integrity, reduced inflammatory infiltration, and partially restored crypt architecture, with the greatest improvement in the TFPS-H group.

AB-PAS staining showed that compared with the MOD group, TFPS-1 treatment partially restored goblet cell abundance and mucus production, especially in the medium- and high-dose groups. These findings suggest that TFPS-1 attenuated DSS-induced colonic histopathological injury and helped preserve the mucus barrier.

#### 3.3.3. Effects on Intestinal Barrier-Related Gene Expression

**Figure 10:**
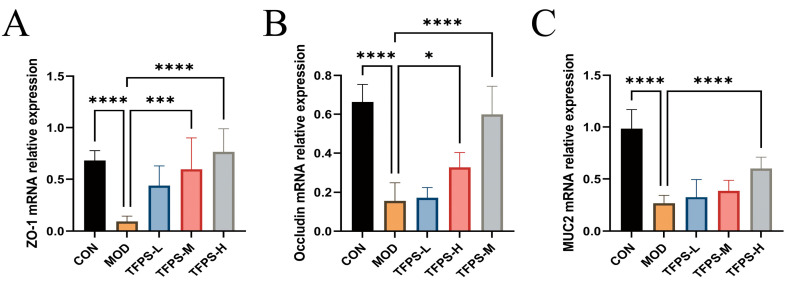
TFPS-1 improved intestinal barrier-related gene expression in DSS-induced experimental colitis mice. (**A**) Relative mRNA expression of ZO-1. (**B**) Relative mRNA expression of occludin. (**C**) Relative mRNA expression of MUC2. Data are presented as mean ± SD (*n* = 6). * *p* < 0.05, *** *p* < 0.001, **** *p* < 0.0001.

To assess the effect of TFPS-1 on barrier-related responses, the transcript levels of ZO-1, occludin, and MUC2 were measured, which are related to tight junction integrity and mucus barrier function. Relative to the control group, DSS treatment led to a marked suppression of these genes’ mRNA levels, as illustrated in Figure 10, indicating downregulation of barrier-related gene expression. In particular, the TFPS-H group showed the greatest increases in ZO-1, occludin, and MUC2 expression. Consistent with the AB-PAS staining results, TFPS-1 treatment was associated with enhanced mucus secretion and upregulated expression of barrier-related genes, indicating a potential improvement in mucus barrier integrity in DSS-induced colitis mice.

#### 3.3.4. Effects on Inflammatory Cytokine Levels

**Figure 11:**
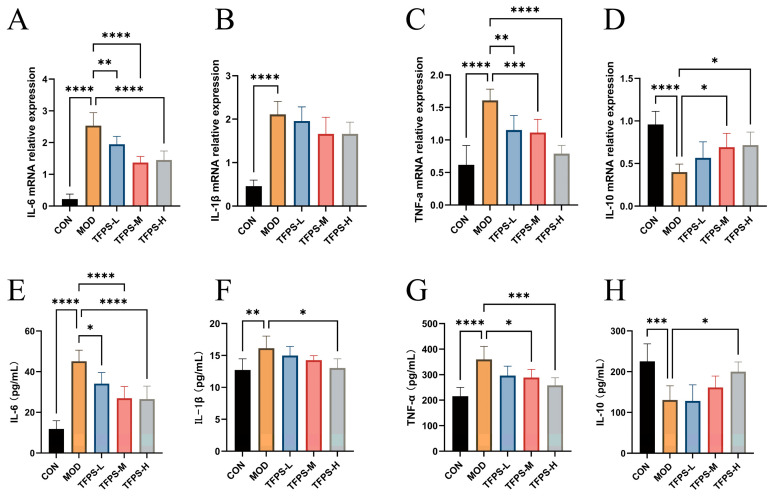
Influence of TFPS-1 on colonic inflammatory cytokine concentrations in mice with DSS-induced colitis. (**A**–**D**) Relative mRNA expression levels. (**E**–**H**) Protein levels. Data are presented as mean ± SD (*n* = 6). * *p* < 0.05, ** *p* < 0.01, *** *p* < 0.001, **** *p* < 0.0001.

As shown in Figure 11, DSS exposure markedly increased the mRNA expression and protein levels of the pro-inflammatory cytokines IL-6, IL-1β, and TNF-α compared with the CON group, while reducing those of the anti-inflammatory cytokine IL-10.

Compared with the MOD group, TFPS-1 treatment decreased both the mRNA expression and protein levels of IL-6, IL-1β, and TNF-α, while increasing IL-10 levels. The regulatory effect was generally more evident in the high-dose group. These findings suggest that TFPS-1 attenuated DSS-induced inflammatory responses in colon tissues, as reflected by improved cytokine profiles.

#### 3.3.5. Effects on Gut Microbiota

**Figure 12:**
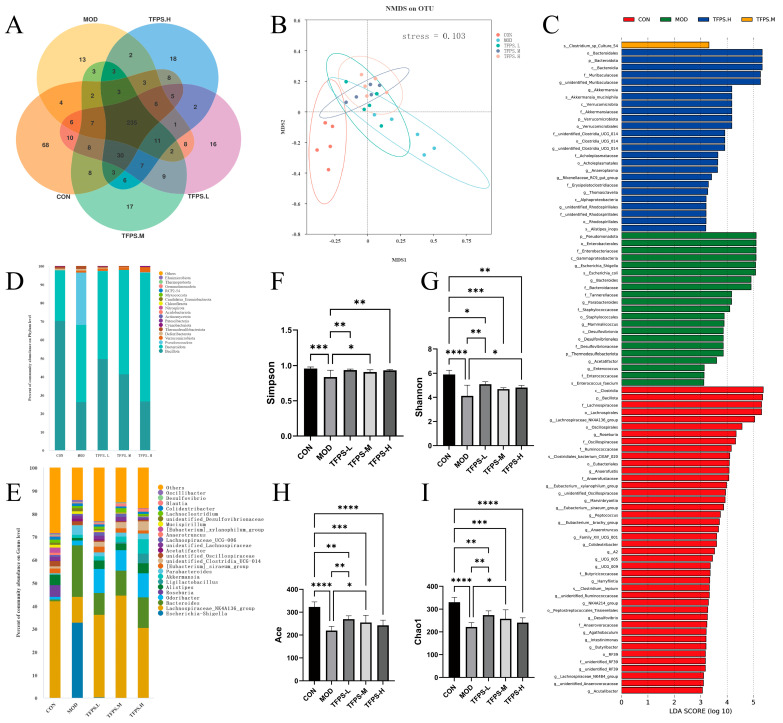
TFPS-1 modulated gut microbiota composition in DSS-induced colitis mice. (**A**) Venn diagram of shared and unique ASVs. (**B**) NMDS analysis of microbial community structure. (**C**) LEfSe-based identification of differentially enriched taxa. (**D**,**E**) Microbial community structure summarized by phylum and genus. (**F**–**I**) Alpha diversity indices, including Simpson, Shannon, ACE, and Chao1. Data are presented as mean ± SD (*n* = 5). * *p* < 0.05, ** *p* < 0.01, *** *p* < 0.001, **** *p* < 0.0001.

The Venn diagram showed that 235 ASVs were shared across all groups, while each group contained group-specific ASVs (Figure 12A). The MOD group showed fewer unique ASVs compared with the CON group, indicating changes in ASV distribution after DSS exposure. TFPS-1-treated groups showed altered ASV profiles compared with the MOD group.

NMDS analysis showed partial separation between the CON and MOD groups, suggesting that DSS altered the overall gut microbiota structure (Figure 12B). TFPS-1-treated groups shifted away from the MOD group and tended to approach the CON group to varying degrees, suggesting partial modulation of DSS-induced changes in microbial community structure.

Alpha diversity analysis showed that Simpson, Shannon, ACE, and Chao1 indices were lower in the MOD group, indicating reduced microbial diversity and richness after DSS exposure (Figure 12F–I). TFPS-1 treatment increased these indices to varying degrees, suggesting partial restoration of microbial diversity and richness.

At the phylum level, DSS exposure altered the relative abundance of dominant bacterial phyla, mainly *Bacillota*, *Bacteroidota*, and *Pseudomonadota* (Figure 12D). Genus-level analysis showed that the DSS challenge elevated the abundance of bacterial groups with pathogenic potential, prominently *Escherichia-Shigella*, while concurrently depleting multiple commensal lineages (Figure 12E). Intervention with TFPS-1 partly reversed these microbiota shifts, evidenced by a suppression of harmful organisms and an enrichment of microbes that support intestinal homeostasis, such as *Akkermansia* and *Muribaculaceae*-related taxa.

LEfSe analysis revealed additional group-specific differences in bacterial taxa (Figure 12C). The MOD group was enriched in *Pseudomonadota*, *Enterobacterales*, *Enterobacteriaceae*, and *Escherichia-Shigella*, whereas the TFPS-H group was enriched in *Akkermansia*, *Muribaculaceae*, and *Bacteroidota*-related taxa. Overall, these results suggest that TFPS-1 partially restored gut microbiota balance by improving microbial diversity and reshaping key bacterial taxa.

## 4. Discussion

By integrating DES-based preparation, structural characterization, and in vivo assessment, this study investigated the intestinal protective potential of *T. fuciformis* polysaccharides. The optimized choline chloride–lactic acid DES system improved TFPS recovery, and the major purified fraction, TFPS-1, was structurally characterized as a low-molecular-weight, glucose-dominant branched glucan. In the DSS-induced colitis model, TFPS-1 attenuated colitis-related injury, accompanied by improved barrier-related gene expression, regulated inflammatory cytokines, and partial restoration of gut microbiota composition. This integrated workflow is relevant because functional evaluation of food-derived polysaccharides requires not only evidence of bioactivity but also efficient extraction and defined material properties [30]. Compared with previous studies that mainly focused on TFPS bioactivity or extraction performance alone [15,16], the present study links DES-based extraction optimization, purified-fraction structural characterization, and disease-relevant in vivo evaluation within the same experimental framework. Nevertheless, the current findings should be interpreted as preclinical evidence from an acute experimental colitis model, rather than as direct evidence of clinical efficacy in UC [31].

The optimized DES-based extraction process produced a higher TFPS yield than hot-water extraction, suggesting that the choline chloride–lactic acid system facilitated polysaccharide release from *T. fuciformis*. This improvement is consistent with the tunable physicochemical properties of DESs, including hydrogen-bonding capacity, polarity, viscosity, and water compatibility, which can enhance mass transfer and weaken interactions between polysaccharides and fungal cell-wall matrices [19,32]. Similar DES-based strategies have been reported for other natural polysaccharides, where solvent composition and extraction parameters influenced both extraction efficiency and physicochemical properties [33,34]. Although DES-based extraction improved TFPS yield in this study, yield improvement alone is insufficient to support practical application. Their safety, biodegradability, and environmental impact are solvent-specific and depend on the components, composition, and concentration of the DES system [35]. In the present study, ethanol precipitation, dialysis, chromatographic purification, and lyophilization were applied to reduce low-molecular-weight impurities, but the absence of residual choline chloride and lactic acid in the final TFPS-1 preparation was not experimentally confirmed by quantitative analysis. Therefore, trace DES residues and their potential effects cannot be completely ruled out, and their potential contribution to the observed biological effects cannot be entirely excluded. The optimized conditions showed acceptable reproducibility in laboratory-scale triplicate validation experiments; however, multiple critical factors remain to be systematically evaluated before any consideration of industrial or food-related applications, including batch-to-batch consistency, solvent recovery and reuse, purification efficiency for complete DES removal, process cost, and large-scale extraction stability [22].

The structural features of TFPS-1 provide a basis for interpreting its UC-related intestinal effects, although they do not directly establish a structure–activity relationship. Its relatively low molecular weight may improve intestinal dispersion and increase accessibility to gut microbiota. Previous studies have suggested that lower-molecular-weight polysaccharides are more readily fermented or degraded by gut bacteria, which provides a possible explanation for the partial recovery of microbial diversity observed in DSS-treated mice after TFPS-1 intervention [36]. The glucose-dominant composition further indicates that TFPS-1 is a glucan-type carbohydrate substrate. Previous studies have shown that *T. fuciformis* polysaccharides can be utilized during fecal fermentation, leading to changes in gut microbiota composition and short-chain fatty acid production [37]. Because short-chain fatty acids are involved in epithelial barrier maintenance and immune homeostasis in IBD, this provides a plausible explanation for the improvements in barrier-related gene expression and inflammatory cytokine profiles observed in the present study [38]. In addition, the coexistence of α-(1→4)- and β-(1→6)-linked glucosyl residues, together with O-6 and O-3 branching sites, may influence microbial degradation and bacterial selectivity. This interpretation is consistent with evidence that glycosidic linkage patterns of fungal polysaccharides can influence microbiota-dependent anti-inflammatory effects in DSS-induced colitis [39]. FTIR and NMR analyses, together with methylation analysis, further confirmed the character of TFPS-1, thereby improving the interpretability of the in vivo results compared with studies using less extensively characterized *T. fuciformis* polysaccharide preparations [16].

In the DSS-induced experimental colitis model, TFPS-1 attenuated the major manifestations of acute colonic injury. Consistent with prior evidence, TFPS can ameliorate DSS-induced colitis by regulating inflammation and epithelial barrier function [15]. DSS-induced colitis is mainly driven by epithelial barrier disruption and subsequent mucosal inflammation, making it suitable for evaluating intestinal protective agents. In the present study, increased mucus secretion and higher mRNA expression of MUC2, ZO-1, and occludin were associated with improved mucus barrier status and upregulation of tight junction-related genes [40,41]. This is biologically relevant because epithelial barrier integrity regulates the interaction between luminal antigens, microbial products, and the mucosal immune system, thereby influencing intestinal immune activation [42]. TFPS-1 also reduced TNF-α, IL-6, and IL-1β while increasing IL-10, indicating a shift toward a less inflammatory mucosal environment. This cytokine pattern is consistent with evidence that an imbalance between pro-inflammatory cytokines and anti-inflammatory cytokines contributes to persistent intestinal inflammation and tissue injury in IBD [43]. In parallel, TFPS-1 partly restored microbial diversity and reduced potentially inflammation-associated taxa such as *Escherichia-Shigella* [44]. TFPS-1 also increased *Akkermansia* and *Muribaculaceae*-related bacteria, which have been associated with intestinal barrier regulation, gut homeostasis, and carbohydrate fermentation in mouse gut ecosystems [45,46]. Together, these data support a coordinated barrier–inflammation–microbiota interpretation of TFPS-1 activity. However, the present study does not establish a direct structure–activity relationship for TFPS-1, because comparisons among TFPS fractions or structural variants with different molecular weights, monosaccharide compositions, linkage patterns, or branching degrees were not performed. Therefore, although the structural characterization of TFPS-1 provides a chemical basis for interpreting its intestinal protective potential, the specific structural features responsible for the observed bioactivity remain to be clarified. In addition, the current biological evidence remains associative rather than causal. Although TFPS-1 was associated with changes in barrier-related genes, inflammatory cytokines, and gut microbiota composition, these endpoints do not provide definitive mechanistic proof. The interpretation should also consider the limitations of the experimental design, including the absence of a positive control drug group, a TFPS-1-only group without DSS exposure, and a residual DES-related control group. Thus, the independent effects of TFPS-1 under normal physiological conditions and the possible influence of trace DES residues cannot be fully evaluated. Accordingly, the observed protective effects should be regarded as promising but preliminary experimental evidence. Future studies should include comparative structure–activity analyses, tight-junction protein validation, intestinal permeability assays, SCFA/metabolomic profiling, microbiota-dependent experiments, positive controls, TFPS-1-only treatment, and residual DES-related controls to clarify the active structural features and mechanisms of TFPS-1.

## 5. Conclusions

This study established an efficient choline chloride–lactic acid DES-based extraction method for *Tremella fuciformis* polysaccharides, achieving a higher TFPS yield than conventional hot-water extraction. The major purified fraction, TFPS-1, was characterized as a low-molecular-weight, glucose-dominant branched glucan mainly containing α-(1→4)- and β-(1→6)-linked glucosyl residues. In a DSS-induced experimental colitis mouse model, TFPS-1 attenuated colitis-related injury, enhanced mucus secretion, upregulated barrier-related gene expression, regulated inflammatory cytokine profiles, and partially restored gut microbiota composition. These findings provide experimental evidence for the preparation, structural characterization, and intestinal protective evaluation of DES-prepared TFPS-1 in an experimental colitis context. Further studies are needed to clarify its potential structure–activity relationship and microbiota-mediated mechanisms, quantitatively assess residual DES levels and their associated safety risks, and rigorously evaluate its feasibility and safety for potential applicability in food systems.

## Figures and Tables

**Table 1:** Factors and levels used in the Box–Behnken design.

Variable	Level		
	−1	0	1
A: extraction temperature (°C)	90	94	98
B: extraction time (min)	150	180	210
C: liquid-to-solid ratio (g/g)	30	35	40
D: LA:ChCl molar ratio	3	3.5	4

**Table 2:** DAI scoring criteria.

Score	Body Weight Loss (%)	Stool Consistency	Fecal Bleeding
0	0	Normal	Negative
1	1–5	Slightly soft stool	Occult blood +^1^
2	5–10	Loose stool	Occult blood ++
3	10–20	Very loose stool	Occult blood +++
4	>20	Watery diarrhea	Gross rectal bleeding

^1^ For fecal bleeding: “+” indicates weakly positive, “++” positive (score 2), “+++” strongly positive.

**Table 3:** Glycosidic linkage composition of TFPS-1 determined by methylation analysis.

Glycosidic Linkage	PMAA Derivative	RT (min)	Relative Molar Ratio (%)
t-Glcp	1,5-di-O-acetyl-2,3,4,6-tetra-O-methyl glucitol	16.791	9.26
→4)-Glcp-(1→	1,4,5-tri-O-acetyl-2,3,6-tri-O-methyl glucitol	20.062	45.12
→6)-Glcp-(1→	1,5,6-tri-O-acetyl-2,3,4-tri-O-methyl glucitol	20.549	39.18
→4,6)-Glcp-(1→	1,4,5,6-tetra-O-acetyl-2,3-di-O-methyl glucitol	23.319	2.25
→3,6)-Glcp-(1→	1,3,5,6-tetra-O-acetyl-2,4-di-O-methyl glucitol	23.484	4.19

Note: PMAA, partially methylated alditol acetate; Glcp, glucopyranosyl residue; t-Glcp, terminal glucopyranosyl residue.

**Table 4:** ^1^H and ^13^C NMR chemical shift assignments of glycosyl residues in TFPS-1.

Residue Code	Glycosyl Residue	Nucleus	Chemical Shifts, δ (ppm)						
			1	2	3	4	5	6a	6b
**G_1,4_**	→4)-α-D-Glcp-(1→	**H**	5.28	3.51	3.84	3.54	3.72	3.65	3.74
		**C**	102.2	74.03	75.68	79.25	73.63	62.92	—
**G_1,4,6_**	→4,6)-α-D-Glcp-(1→	**H**	5.25	3.48	3.89	3.53	3.71	3.77	—
		**C**	102.5	74.00	75.82	79.25	74.00	70.43	—
**G_t_**	α-D-Glcp-(1→	**H**	4.85	3.47	3.90	3.55	3.74	3.85	—
		**C**	101.1	74.00	75.79	71.47	73.74	63.17	—
**G_1,6_**	→6)-β-D-Glcp-(1→	**H**	4.41	3.21	3.39	3.35	3.52	3.74	4.10
		**C**	105.5	75.53	78.10	72.10	77.32	71.33	—
**G_1,3,6_**	→3,6)-β-D-Glcp-(1→	**H**	4.44	3.39	3.64	3.48	3.55	3.74	4.10
		**C**	105.5	75.53	86.77	70.80	77.46	71.33	—
**G′_t_**	β-D-Glcp-(1→	**H**	4.63	3.25	3.38	3.29	3.35	3.61	3.81
		**C**	105.4	75.94	78.10	72.10	78.10	63.17	—

## Data Availability

The raw 16S rRNA sequencing data generated in this study have been deposited in the NCBI Sequence Read Archive under BioProject accession number PRJNA1477853. Other data supporting the findings of this study are included in the article and Supplementary Materials or are available from the corresponding author upon reasonable request.

## Supplementary Materials

The following supporting information can be downloaded at https://www.mdpi.com/article/10.3390/foods15122207/s1: Table S1. Response surface test design and results; Table S2. The regression model of variance analysis results; Table S3. Primers for RT-qPCR; Figure S1. Relative abundance of selected differential bacterial taxa in DSS-induced colitis mice.
