# Supplementary material for: Deep Eutectic Solvent-Based Extraction Optimization, Structural Characterization, and Alleviating Effects of Tremella fuciformis Polysaccharides on Ulcerative Colitis
Source: Foods. 2026 Jun 18;15(12):2207. doi: 10.3390/foods15122207 (PMC13298562; doi:10.3390/foods15122207)
Supplement: Supplementary file 1 [file foods-15-02207-s001.zip › foods-4361787-supplementary.pdf]

Supplementary Materials

Table S1. Response surface test design and results.

Number	A（°C）	B（min）	C (g/g)	D	Yield（%）
1	98	210	35	4	31.88
2	94	180	40	3	30.18
3	94	150	35	3	30.15
4	98	150	35	4	28.51
5	98	180	40	4	28.97
6	94	180	35	4	32.21
7	90	150	35	4	26.92
8	94	210	40	4	29.89
9	90	180	35	4	26.65
10	94	150	30	4	29.10
11	94	180	30	3	30.94
12	94	150	40	4	28.82
13	94	180	40	4	27.91
14	90	180	40	4	24.81
15	94	210	35	4	32.00
16	94	210	30	4	31.63
17	94	210	35	3	32.02
18	98	180	30	4	30.19
19	98	180	35	4	29.64
20	94	180	35	4	32.69
21	94	180	35	4	32.69
22	98	180	35	3	31.20
23	94	180	35	4	32.70
24	94	150	35	4	27.70
25	90	180	30	4	26.18
26	90	210	35	4	26.22
27	94	180	30	4	30.35
28	90	180	35	3	26.31
29	94	180	35	4	32.30

Table S2. The regression model of variance analysis results.

**Source**	**Sum of Squares**	df	Mean Square	F-value	p-value	Significance
Model	148.60	14	10.61	56.52	< 0.0001	Significant
A-Extraction temperature	45.24	1	45.24	240.89	< 0.0001	
B-Extraction time	12.90	1	12.90	68.67	< 0.0001	
C-Liquid-to-solid ratio	5.08	1	5.08	27.06	0.0001	
D-LA-to-ChCl Molar ratio	3.58	1	3.58	19.04	0.0006	
AB	4.14	1	4.14	22.05	0.0003	
AC	0.0056	1	0.0056	0.0300	0.8651	
AD	0.9025	1	0.9025	4.81	0.0458	
BC	0.5329	1	0.5329	2.84	0.1142	
BD	1.48	1	1.48	7.86	0.0141	
CD	0.7056	1	0.7056	3.76	0.0730	
A^2	64.95	1	64.95	345.85	< 0.0001	
B^2	6.41	1	6.41	34.15	< 0.0001	
C^2	19.37	1	19.37	103.15	< 0.0001	
D^2	6.08	1	6.08	32.37	< 0.0001	
Residual	2.63	14	0.1878			
Lack of fit	2.39	10	0.2395	4.08	0.0938	Not significant
Pure error	0.2347	4	0.0587			
**Cor Total**	151.23	28				

Table S3. Primers for RT-qPCR.

gene	5′ to 3′	5′ to 3′
β -Actin	GCTCTGGCTCCTAGCACCAT	GCCACCGATCCACACAGAGT
TNF-α	CAGGCGGTGCCTATGTCTC	CGATCACCCCGAAGTTCAGTAG
IL-6	CTGCAAGAGACTTCCATCCAG	AGTGGTATAGACAGGTCTGTTGG
IL-1β	TTCAGGCAGGCAGTATCACTC	GAAGGTCCACGGGAAAGACAC
IL-10	CTTACTGACTGGCATGAGGATCA	GCAGCTCTAGGAGCATGTGG
ZO-1	GCCTTGAACTTTGACCTCTGC	GAAATCGTGCTGATGTGCCA
Occludin	CCGGCCGCCAAGGTTC	GCTGATGTCACTGGTCACCTA
MUC2	ATGCCCACCTCCTCAAAGAC	GTAGTTTCCGTTGGAACAGTGAA

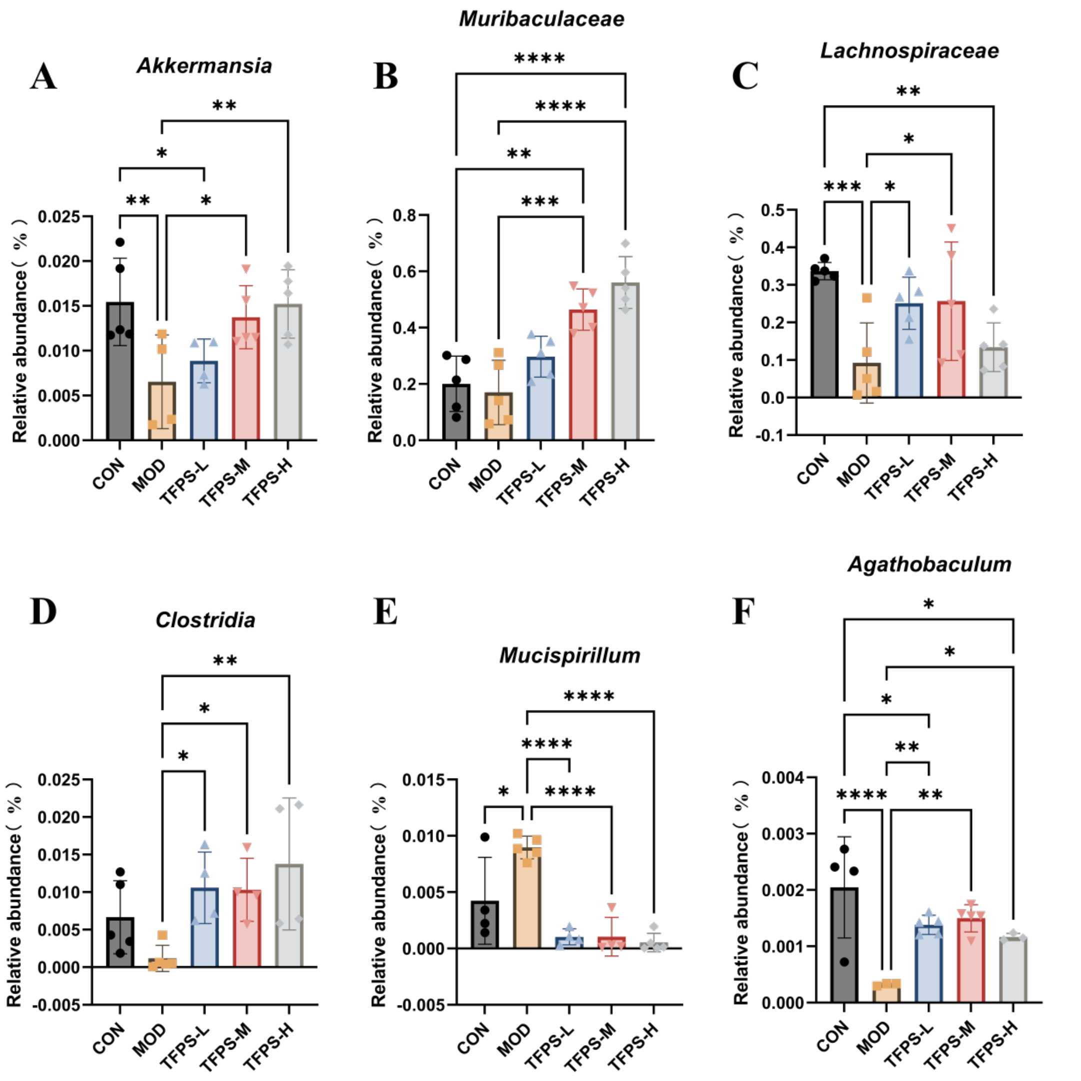

Figure S1. Relative abundance of selected differential bacterial taxa in DSS-induced colitis mice. (A) Akkermansia; (B) Clostridia; (C) Lachnospiraceae; (D) Muribaculaceae; (E) Mucispirillum; and (F) Agathobaculum. Data are presented as mean ± SD (n = 5). * $p < 0.05$, ** $p < 0.01$, *** $p < 0.001$, and **** $p < 0.0001$ indicate significant differences between groups.
